# Heavy Metals’ Effect on Susceptibility to Attention-Deficit/Hyperactivity Disorder: Implication of Lead, Cadmium, and Antimony

**DOI:** 10.3390/ijerph15061221

**Published:** 2018-06-10

**Authors:** Min-Jing Lee, Miao-Chun Chou, Wen-Jiun Chou, Chien-Wei Huang, Ho-Chang Kuo, Sheng-Yu Lee, Liang-Jen Wang

**Affiliations:** 1 Department of Child and Adolescent Psychiatry, Kaohsiung Chang Gung Memorial Hospital, Kaohsiung 833, Taiwan; 8035c@cgmh.org.tw (M.-J.L.); ks7968@cgmh.org.tw (M.-C.C.); wjchou@cgmh.org.tw (W.-J.C.); 2 Chang Gung University College of Medicine, Kaohsiung 833, Taiwan; erickuo48@yahoo.com.tw; 3 Division of Nephrology, Department of Medicine, Kaohsiung Veterans General Hospital, and National Yang-Ming University, School of Medicine, Kaohsiung 813, Taiwan; cwhuang0824@gmail.com; 4 Department of Pediatrics and Kawasaki Disease Center, Kaohsiung Chang Gung Memorial Hospital, Kaohsiung 833, Taiwan; 5 Department of Psychiatry, Kaohsiung Veterans General Hospital, Kaohsiung 813, Taiwan; shirleylee.ncku@gmail.com; 6 Department of Psychiatry, College of Medicine and Hospital, National Cheng Kung University, Tainan 704, Taiwan

**Keywords:** ADHD, lead, antimony, clinical symptoms, intelligence quotient

## Abstract

*Background:* Heavy metals are known to be harmful for neurodevelopment and they may correlate to attention deficit/hyperactivity disorder (ADHD). In this study, we aim to explore the relationships between multiple heavy metals (manganese, lead, cadmium, mercury, antimony, and bismuth), neurocognitive function, and ADHD symptoms. *Methods:* We recruited 29 patients with ADHD inattentive type (ADHD-I), 47 patients with ADHD hyperactivity/impulsivity type (ADHD-H/I), and 46 healthy control children. Urine samples were obtained to measure the levels of the aforementioned heavy metals in each child. Participants’ cognitive function and clinical symptoms were assessed, respectively. *Results:* We found ADHD-H/I patients demonstrated the highest antimony levels (*p* = 0.028), and ADHD-I patients demonstrated the highest cadmium levels (*p* = 0.034). Antimony levels were positively correlated with the severity of ADHD symptoms that were rated by teachers, and cadmium levels were negatively correlated with the Full Scale Intelligence Quotient. Lead levels were negatively correlated with most indices of the Wechsler Intelligence Scale for Children–Fourth Edition (WISC-IV), but positively correlated with inattention and hyperactivity/impulsivity symptoms (*p* < 0.05). *Conclusion:* Lead, cadmium and antimony were associated with susceptibility to ADHD and symptom severity in school-age children. Eliminating exposure to heavy metals may help to prevent neurodevelopmental disorders in children.

## 1. Introduction

Attention deficit/hyperactivity disorder (ADHD), which is characterized by inattention, impulsivity, and hyperactivity, is among the most common psychiatric disorders occurring in children [1,2]. The American Psychiatry Association (APA) has estimated ADHD prevalence in 3% to 7% of children in the United States [3], and has indicated that these children are at a high risk of developing emotional and behavioral disorders [4]. Currently, the exact etiology of ADHD continues to be unclear. Excessive metals are detrimental to neurodevelopmental processes and have neurotoxic effects that impair cognitive function. Therefore, heavy metals may be involved in susceptibility to ADHD.

Previous studies have reported that environmental toxicants, especially lead (Pb) and mercury (Hg), contribute to the risk of ADHD [5]. A meta-analysis that was conducted by Goodlad et al. found that Pb exposure was associated not only with inattention and hyperactivity/impulsivity symptoms, but also intelligence among children and adolescents, with a small effect size (r= 0.13–0.16) [6]. In another meta-analysis, Yoshimasu found that environmental Hg exposure was associated with a 1.7-fold increased risk of autism spectrum disorders (ASD) and a 1.6-fold increased risk of ADHD [7]. Furthermore, one systematic review article reported that a 50% increase of manganese (Mn) levels in hair might be associated with a 0.7-point decrease in the intelligence quotient (IQ)of children aged 6–13 years [8]. 

Hjortenkrans et al. reported that brake linings was an important source of zinc (Zn), cooper (Cu), and antimony (Sb) emissions and tires are one of the main sources of Zn and cadmium (Cd) emissions [9]. Cd is broadly used in the production of rechargeable nickel-cadmium batteries, in the coloring of plastics, and as the stabilizer of polyvinylchloride (PVC) and other related polymers. Studies investigating the association between cadmium and ADHD have been limited, and their findings are mixed. The National Health and Nutrition Examination Survey (NHANES) in Korea showed a weak correlation between Cd levels in blood and attention deficit disorder [10], and a positive link between Cd in children’s urine and learning disabilities and the need for special education among school-age children [11]. However, other studies have had conflicting results [12,13]. Szkup-Jablonska reported no negative effect of cadmium on the functioning of children with ADHD, while Kim’s study was unable to find a correlation between Cd or Hg exposure and ADHD.

Sb is frequently used as a catalyst in the polycondensation reaction to produce polyethylene terephthalate (PET) and it may leach from PET and recycled bottles into drinking water. Humans can be exposed to Sb by breathing air, drinking water, and eating foods that contain it, as well as by skin contact with substances that contain it. The effect of Sb on human heath has not yet been fully established. Only one study has reported no significant association between prenatal exposure to Sb and cognitive function at the age of four years [14]. Bismuth (Bi) salt is commonly used to treat gastrointestinal discomfort. Some Bi poisoning events among children have been related to Bi overdoses to treat diarrhea. However, the roles of Sb and Bi in ADHD presentation remain unknown.

Based on clinical characteristics, the Diagnostic and Statistical Manual of Mental Disorders, Fourth Edition, Text Revision (DSM-IV-TR) classified ADHD into three subtypes (i.e., predominant inattentive, predominant hyperactivity, and combined type) [15]. Previous studies have proposed that clinical presentations between ADHD subtypes may be the result of different pathogenesis. So far, few studies have examined the association between environmental toxicants and ADHD subtypes. Nevertheless, some studies have shown that Pb exposure is associated with children’s hyperactivity/impulsivity behavior, but not inattentiveness [5,16,17,18]. Whether profiles of heavy metals besides Pb vary between different ADHD subtypes and healthy controls is still poorly understood. We carried out a case control study to investigate the possible differences in the urinary levels of Mn, Pb, Cd, Hg, Sb, and Bi between patients with different ADHD subtypes and healthy controls, as well as whether these heavy metal levels may influence the psychopathology and neurocognitive functions of ADHD.

## 2. Material and Methods

### 2.1. Study Participants

Our research protocol was approved by the Institutional Review Board at Chang Gung Hospital in Taiwan. We recruited patients with ADHD who were treated in the outpatient Department of Child Psychiatry at Chang Gung Children’s Hospital in Taiwan for this cross-sectional study. We obtained written informed consent from all of the participants or their guardians. The inclusion criteria consisted of the following: (a) clinical diagnosis of ADHD by a senior child psychiatrist based on the DSM-IV-TR through structured interviews using the Chinese version of the Schedule for Affective Disorders and Schizophrenia for School-Age Children, epidemiologic version (K-SADS-E) [19]; (b) between 6 and 16 years old; and, (c) a new diagnosis of ADHD in a drug-naïve patient or one with an existing diagnosis but that had not used an ADHD medication for at least six months. Patients with a history of comorbid pervasive developmental disorder, intellectual disability, major depressive disorder, bipolar disorder, psychosis, epilepsy, or brain injury were excluded. 

The control subjects (children without ADHD) consisted of healthy children from communities surrounding Kaohsiung Chang Gung Memorial Hospital or children that were suffering from upper respiratory tract infection (URI) whose symptoms were currently in remission. We excluded any patients with major psychiatric disorders (such as intellectual disabilities, autism spectrum disorder, bipolar disorders, major depressive disorders, psychotic disorders, substance dependence, epilepsy, or severe head trauma) or major physical illnesses (such as genetic, metabolic, or infectious conditions). 

In total, 46 healthy control children, 48 patients with ADHD inattentive type (ADHD-I), and 65 patients with ADHD hyperactivity/impulsivity type (ADHD-H/I) were recruited for this study. Because maturation processes and education effects could be diverse with age, we selected patents <10 years old into analyses.

### 2.2. Urinary Toxic Metal Screening Test

A urine sample (10 mL) was collected from each child and sent to the Chang Gung Memorial Hospital laboratory. We measured metal concentrations, including Mn, Pb, Cd, Hg, Sb, and Bi, using inductively coupled plasma mass spectrometry (ICP/MASS) and mass spectrometry in order to simultaneously measure the concentration of various heavy metals. ICP/MASS uses argon to generate high temperature plasma (6000K) in order to atomize and then ionize the heavy metal compounds. The mass spectrometer then directed the metal ions and analyzed each metal’s content based on its mass.

### 2.3. Clinical Measurements

Each ADHD patient was interviewed by a senior psychiatrist using the K-SADS-E diagnostic tool. An experienced child psychologist conducted the Wechsler Intelligence Scale for Children–Fourth Edition (WISC-IV). Furthermore, the Swanson, Nolan, and Pelham Version IV Scale (SNAP-IV) parent form and SNAP-IV teacher form were completed by each patient’s parents and a teacher, respectively.

The K-SADS-E is a semi-structured diagnostic interview that is designed to evaluate current and past episodes of psychopathology in children and adolescents, according to the criteria of the DSM-III-R and DSM-IV [19]. The K-SADS-E involves interviewing the parent(s) and the child and determining summary ratings that include all the sources of information. The validity and reliability of the Chinese version of K-SADS-E has been previously established in Taiwan [20].

The Chinese version of the WISC-IV is an individually administered and norm-referenced instrument developed to measure the intelligence of children aged from 6 to 16 years old [21]. The WISC-IV contains 10 core and five supplemental subtests. The core subtests form the following four factor indexes: the Verbal Comprehension Index (VCI), the Perceptual Reasoning Index (PRI), the Working Memory Index (WMI), and the Processing Speed Index (PSI). The Full Scale Intelligence Quotient (FSIQ) consists of the 10 core subtests. The factor indexes and FSIQ each have a population mean of 100 and a standard deviation of 15 [22].

The SNAP-IV is a 26-item questionnaire for evaluating ADHD symptoms and severity, and it needs to be completed by parents or teachers [23]. The 26 items include 18 for ADHD symptoms (nine for inattentive and nine for hyperactive/impulsive) and eight for oppositional defiant disorder (ODD) symptoms as defined in the DSM-IV. Each item is scored from 0 to 3 on a Likert scale. The Chinese version of the SNAP-IV parent form [24] and the SNAP-IV teacher form [25] have been reported to have satisfactory levels of reliability and concurrent validity. 

### 2.4. Statistical Analysis

Data were analyzed using the statistical software package SPSS, version 16.0 (SPSS Inc., Chicago, IL, USA). Variables are presented as either the mean (standard deviation) or frequency. Two-tailed *p*-values of <0.05 were considered statistically significant.

Categorical variables among the healthy control group, ADHD-I group, and ADHD-H/I group were compared using the chi-square test. We adopted one-way analysis of variance (ANOVA) to compare the continuous variables among groups, followed by a post-hoc Least Significant Difference (LSD) test. Levels of heavy metals in the urine samples demonstrated a significant level of positive skewness. Therefore, the Kruskal-Wallis test was adopted to compare continuous variables among the three groups. Spearman’s correlation was performed to analyze the relationships between heavy metal levels, neuropsychological functions, and ADHD clinical symptoms.

## 3. Results

Our study sample consisted of 46 healthy control children (mean age 8.1 years), 29 ADHD-I children (mean age 8.0 years), and 47 ADHD-H/I children (mean age 7.7 years). Among these groups (Table 1), ADHD-H/I had the highest proportion of boys (85.1%). When compared to the ADHD-I group, the healthy controls demonstrated superior performance in all the indexes of the WISC-IV (FSIQ, PRI, WMI, and PSI). As compared to the ADHD-H/I group, the healthy controls demonstrated superior performance in FSIQ, WMI, and PSI scores. Of all the dimensions of ADHD clinical symptoms (parent-rated and teacher-rated inattention scores, hyperactivity/impulsivity scores, and oppositional scores of the SNAP-IV), ADHD-H/I exhibited the highest severity, followed by ADHD-I; healthy controls had the lowest severity.

**Figure 1:**
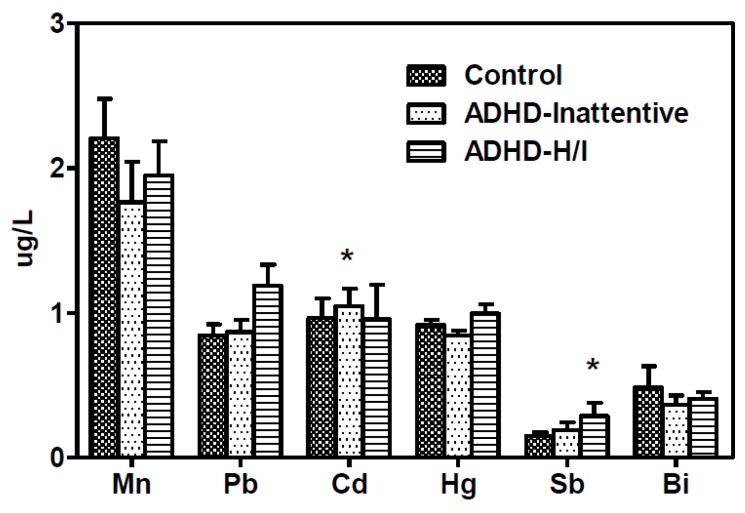
Urinary levels of manganese (Mn), lead (Pb), cadmium (Cd), mercury (Hg), antimony (Sb), and bismuth (Bi) of patients with ADHD inattentive type (ADHD-I), patients with ADHD hyperactivity/impulsivity type (ADHD-H/I), and healthy control subjects. Children with ADHD-I demonstrated the highest Cd (*p* = 0.034) levels, and children with ADHD-H/I demonstrated the highest Sb levels (*p* = 0.028).

Figure 1 illustrates the urinary levels of six heavy metals across the three participant groups. When compared to the healthy controls and the ADHD-H/I group, children with ADHD-I demonstrated the highest Cd (*p* = 0.034) levels. Compared to the healthy controls and ADHD-I group, children with ADHD-H/I demonstrated the highest Sb levels (*p* = 0.028). However, we observed no significant differences among the groups for Mn (*p* = .485), Pb (*p* = 0.125), Hg (*p* = 0.132), or Bi (*p* = 0.434).

Table 2 summarizes the relationships between heavy metal levels, neuropsychological functions, and ADHD clinical symptoms among all of the participants (*n* = 122). We found that Pb levels negatively correlated with FSIQ, VCI, WMI, and PSI of the WISC-IV and positively correlated with the inattention, hyperactivity/impulsivity, and oppositional scores rated by parents and teachers, respectively. Cd levels were negatively correlated with FSIQ scores, while Sb levels were positively correlated with inattention, hyperactivity/impulsivity, and oppositional scores that were rated by teachers. Levels of Hg were positively correlated to hyperactivity/impulsivity scores rated by parents. Levels of Mn and Bi were observed to not have a significant association with ADHD clinical symptoms or cognitive functions.

## 4. Discussion

In the present study, we investigated the exposure of such metals as Mn, Pb, Cd, Hg, Sb, and Bi and its relationship to the specificity of ADHD symptomatology, measuring severity and cognitive function with WISC-IV. We found that Pb levels were positively correlated with ADHD symptomatology, including inattention, hyperactivity, and impulsivity. From previous studies, we know that Pb interrupts the dopamine pathway, which is one of the major neurotransmitter pathways that is involved in ADHD, resulting in dopaminergic neuron damage and the disruption of the homeostasis of calcium-dependent neurotransmitters [5]. Previous research has shown a positive association between Pb and ADHD symptoms or ADHD diagnosis [5,6,12,17,18,26,27]. Our findings support this point of view, and it implies that Pb plays a role in the attention process and behavior control. Nevertheless, our cross-sectional study could not determine the causality of Pb and ADHD. For example, socio-economic status (SES) simultaneously influences the risk of Pb exposure and ADHD, and thus may lead to a significant association between Pb levels and ADHD. In addition, epidemiological studies showed that the prevalence of ADHD were not declined, and even increased [28], in the past decades, through the phasing out lead based petrol had become common public health policy in most countries. Many other factors interfering the neurodevelopment, including individual differences, genetic heredity, socioenvironmental factors, and educating style have to be considered to be related ADHD symptoms. Therefore, lead may not be the only environmental variable that is associated with ADHD, and the interactive effects between multiple environmental neurotoxicants warranted further clarification.

Our study also suggested that Pb levels were negatively correlated with FSIQ, VCI, WMI, and PSI of the WISC-IV. Such cognitive function impairment may result from Pb-induced directed cell death and the interruption of intracellular biological activities [29]. Our finding agrees with many previous studies that have reported an association between Pb exposure and reduced intellectual functioning [18,30,31,32,33,34,35]. A previous study that was conducted by Surkan et al. examined the relationship between neuropsychological function and Pb levels, in which they found an inverse relationship between Pb levels and attention, working memory, and FSIQ, as well as that verbal IQ was more negatively affected than performance IQ [32]. Hong et al. also reported that FSIQ and verbal IQ had a negative correlation with Pb levels [4]. However, the causal relationship between Pb, IQ, and ADHD is still inconclusive. Goodman et al. and Nigg et al. suggested that IQ represents a weak mediating effect between Pb level and ADHD [6,17]. On the other hand, Hong et al. proposed that Pb exposure had independent effects on both intelligence and ADHD [18]. Various confounding factors, such as socio-economic status, parents’ education, and other environmental chemicals may also interfere with the causal relationship. Our present analysis may not provide a clear determination of causal direction. Nonetheless, the detrimental effects of Pb to cognitive function were well-established, the public health policy for reduction the environmental Pb, such as lead painting and lead pipe, is urgent.

Another interesting finding in our study is that the highest Sb levels presented in the ADHD-H/I group and were lower in both the ADHD-I group and the healthy control group. Furthermore, Sb levels were positively correlated with the inattention, hyperactivity/impulsivity, and oppositional scores rated by teachers. Other experimental studies and case reports have suggested that Sb may cause peripheral neurotoxicity [36], cerebellar ataxia [37], and genotoxicity [38,39]. However, research is lacking with regard to the toxic effect of Sb on neurocognition. One meta-analysis linked Sb levels to autism, but was lacking neurobiological evidence [40]. Our findings serve as a reminder that clinicians must pay particular attention to the possible effect of Sb on ADHD. In the future, further research is needed to investigate the relationship between Sb and ADHD.

When compared to the healthy controls and the ADHD-H/I group, children with ADHD-I demonstrated the highest Cd levels. We also found in our study that levels of Cd were negatively correlated with FSIQ scores. A prospective study of 1305 subjects identified significantly inverse associations between urinary Cd levels and VIQ and FSIQ [41]. Another cross-sectional study also reported that Cd levels in urine had a negative association with VIQ in 6 to 9-year-old children [42]. Cd-induced neurotoxicity is complex and has been associated with both biochemical changes of the cell and functional changes of the central nervous system [43]. However, the main target brain region of Cd-mediated toxicity remains undetermined [44,45,46]. Our findings encourage further exploration on the effects of Cd with regard to the neurocognitive function of verbal comprehension.

In the present study, we also found that the levels of Hg were positively correlated to hyperactivity/impulsivity scores rated by parents. Hg exerts the detrimental effect on enzymes, cellular membrane function, and neurotransmitter levels [47]. A meta-analysis conducted by Yoshimasu demonstrated the significant association between environmental exposure to Hg and ADHD [7]. It is noteworthy that a majority of those studies assessed their outcomes with miscellaneous neurodevelopmental indicators, such as ADHD symptoms rated by Conner’s scale, rather than using psychiatric diagnostic categories of ADHD, as defined by DSM-IV [48,49,50]. Our finding was similar with prior data and further highlight the need for understanding the relationship between Hg and ADHD symptomatology.

This study has certain limitations. First, the cross-sectional nature of the study design and the single measurement of Pb and the other environmental chemicals were not sufficient to establish a definite causal relationship. In addition, the range of the half-life of these heavy metals was widely dispersed. For example, Sb had the shortest half-life with 95 hours after exposure, but Cd had the longest half-life for decades. The single measurement of urine sample may reflect recent exposure. However, it is difficult to clarify the effect of heavy metals during the critical period of neurodevelopment. Second, using urine samples to measure chemicals may be related to more individual variations, especially in Pb. Furthermore, urinary Pb levels are less sensitive in the low range of exposures (i.e., <10 μg/dL) [51]. As for Mn, the optimal measurement for biological monitoring is still controversial [52,53,54]. Nevertheless, urinary Hg, Cd, and Sb seem to be good markers for exposure [55,56]. Therefore, we used urine samples as a compromised measurement for monitoring the various metals. Third, we did not evaluate some potentially confounding variables, such as children’s school types, school marks, particular learning disability, family’s SES, parents’ IQ, and other environmental exposure. With regard to SES, which have been considered as a potential confounder of the association between lead exposure and children’s neurodevelopment in early studies [57]. Failure to control for the aforementioned factors was a major limitation of the study. Finally, the subjects were only obtained from one site with a small sample size, which limited the ability to extend our findings to other populations. Our control subjects were not recruited through random sampling, the study sample might not represent the community population. This referral bias may also influence the results of this study. 

## 5. Conclusions

Our study’s strength came from measuring various metals, as well as demonstrating the effect in subjects with a formal ADHD diagnosis that is based on DSM-IV-TR criteria. Our findings indicate metals’ relationship to susceptibility to ADHD, especially for Pb, Cd, and Sb. Children’s neurocognitive function is particularly correlated to Pb levels. These findings suggest that children should avoid heavy metal exposure and prevent the adverse effect of metals on children’s neurodevelopment. However, Taiwan is a cramped and densely populated country. The demarcation of the cultivated field, industrial area, and the residential area was often not clear. Therefore, it is crucial for legislating public health policy for reduction of the environmental heavy metals exposure.

## Figures and Tables

**Table 1:** Characteristics of the healthy control children, patients with attention deficit/hyperactivity disorder (ADHD) inattentive type (ADHD-I), patients with ADHD hyperactivity/impulsivity type (ADHD-H/I).

Variables	Healthy Control (*n* = 46)	ADHD-I (*n* = 29)	ADHD-H/I (*n* = 47)	Statistic Value	*p*-Value	Post-hoc Test
Gender, *n* (%)				18.113	<0.001	
Boy	31 (67.4)	11 (37.9)	40 (85.1)			
Girl	15 (32.6)	18 (62.1)	7 (14.9)			
Age (years)	8.1 ± 1.2	8.0 ± 1.0	7.7 ± 1.0	1.860	0.160	
Height (cm)	129.4 ± 9.8	127.7 ± 8.1	128.3 ± 8.6	0.367	0.694	
Weight (kg)	29.1 ±8.7	28.8 ± 8.0	29.3 ± 9.6	0.031	0.969	
WISC-IV						
Full Scale Intelligence Quotient	108.9 ± 13.9	98.9 ± 10.4	102.7 ± 8.4	7.649	0.001	C > I, C > H
Verbal Comprehension Index	108.1 ± 12.0	103.3 ± 9.5	106.6 ± 10.8	1.724	0.183	
Perceptual Reasoning Index	108.2 ± 16.7	98.8 ± 14.3	102.9 ± 11.1	4.149	0.018	C > I
Working Memory Index	107.8 ± 12.8	100.8 ± 11.6	100.9 ± 8.8	5.619	0.005	C > I, C > H
Processing Speed Index	102.9 ± 11.8	92.3 ± 8.3	96.6 ± 8.4	11.037	<0.001	C > I, C > H
Clinical measures						
SNAP-IV parent form (I)	6.3 ± 5.7	15.1 ± 5.6	17.5 ± 4.7	55.559	<0.001	H > C, I > C
SNAP-IV parent form (H)	5.1 ± 5.7	10.5 ± 4.8	17.5 ± 5.4	62.050	<0.001	H > I > C
SNAP-IV parent form (O)	5.5 ± 5.3	10.3 ± 5.5	13.7 ± 6.1	24.117	<0.001	H > I > C
SNAP-IV teacher form (I)	5.7 ± 5.2	12.4 ± 7.0	16.1 ± 5.0	40.790	<0.001	H > I > C
SNAP-IV teacher form (H)	3.8 ± 3.7	7.2 ± 5.5	15.2 ± 5.7	62.738	<0.001	H > I > C
SNAP-IV teacher form (O)	2.3 ± 2.6	4.8 ± 5.2	10.3 ± 5.5	37.640	<0.001	H > I > C

Note: Data are expressed as mean ± SD or *n* (%). WISC-IV, the Wechsler Intelligence Scale for Children–Fourth Edition; SNAP-IV, the Swanson, Nolan, and Pelham Version IV Scale; I, inattention scores; H, hyperactivity/impulsivity scores; O, oppositional scores. Statistical values are expressed as F-value or χ^2^. Acronym in post-hoc test: C, Healthy Control; I, ADHD-I; H, ADHD-H/I.

**Table 2:** Relationship between urinary levels of heavy metals, ADHD clinical symptoms, and neuropsychological functions among all participants (*n* = 122).

Variables	Mn	Pb	Cd	Hg	Sb	Bi
WISC-IV						
Full Scale Intelligence Quotient	−0.116	−0.294 **	−0.185 *	−0.057	−0.086	−0.052
Verbal Comprehension Index	−0.135	−0.273 **	−0.162	0.025	−0.145	−0.115
Perceptual Reasoning Index	−0.006	−0.094	−0.155	−0.002	0.024	0.005
Working Memory Index	−0.050	−0.393 ***	−0.158	−0.100	−0.122	−0.088
Processing Speed Index	−0.133	−0.262 **	−0.097	0.033	−0.091	−0.024
Clinical measures						
SNAP-IV parent form (I)	−0.079	0.231 *	−0.027	0.058	0.116	0.139
SNAP-IV parent form (H)	−0.093	0.220 *	−0.095	0.199 *	0.060	0.147
SNAP-IV parent form (O)	−0.049	0.186 *	−0.067	0.160	0.004	0.161
SNAP-IV teacher form (I)	0.017	0.242 **	−0.086	0.130	0.273 **	−0.024
SNAP-IV teacher form (H)	0.027	0.214 *	−0.148	0.055	0.317 ***	0.005
SNAP-IV teacher form (O)	0.000	0.214 *	−0.142	0.095	0.196 *	0.026

Note: Data are expressed as Spearman’s correlation coefficient; * *p* < 0.05, ** *p* < 0.01, *** *p* < 0.001. WISC-IV, the Wechsler Intelligence Scale for Children–Fourth Edition; SNAP-IV, the Swanson, Nolan, and Pelham Version IV Scale; I, inattention scores; H, hyperactivity/impulsivity scores; O, oppositional scores.
